# Public health round-up

**DOI:** 10.2471/BLT.23.010123

**Published:** 2023-01-01

**Measles coverage down**

A child being vaccinated in Al Hol displacement camp, north-east Syria, on 25 October 2022. In 2021, nearly 40 million children missed a measles vaccine dose, continuing a steady decline in coverage since the beginning of the COVID-19 pandemic.

**Figure:**
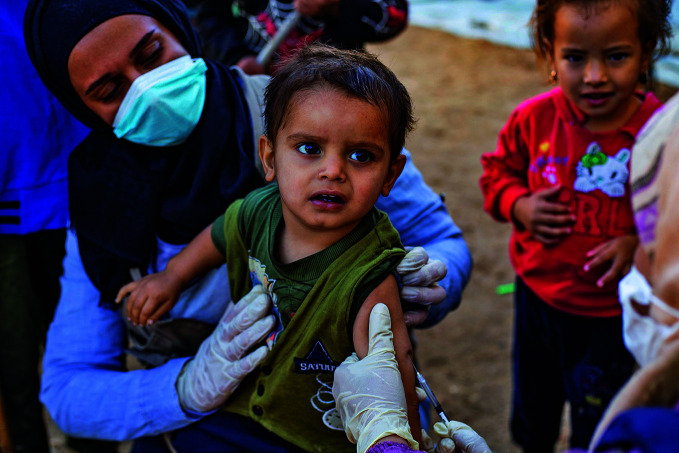
UNICEF/Delil-Souleiman

## Increasing antimicrobial resistance

A high proportion (> 50%) of bacteria that are frequently the cause of bloodstream infections in hospitals, such as *Klebsiella pneumoniae* and *Acinetobacter spp*. are exhibiting antimicrobial resistance (AMR).

This is according to the World Health Organization’s (WHO) fifth *Global antimicrobial resistance and use surveillance system report* which was released on 9 December, summarizing data on AMR and antimicrobial consumption reported by 87 countries.

To treat such infections, clinicians use last-resort antibiotics, such as carbapenems. However, as the report notes, around 8% of bloodstream infections caused by *K. pneumoniae* are also resistant to carbapenems.

The report also reveals increasing resistance in several bacteria causing common infections, among them *Escherichia coli*, the most common pathogen in urinary tract infections.

Although most resistance trends have remained stable over the past 4 years, bloodstream infections due to resistant *E. coli* and *Salmonella spp*. and resistant gonorrhoea infections increased by at least 15% compared to rates reported in 2017.

https://bit.ly/3W1Ys91

## Tackling antimicrobial resistance

In related news, participants at the Third Global High-Level Ministerial Conference on Antimicrobial Resistance which took place in Muscat, Oman, on 24–25 November agreed to reduce the total amount of antimicrobials used in agrifood systems by at least 30% by 2030, preserve critically important antimicrobials for human medicine, and ensure that “Access” antibiotics (those that are affordable, safe and have a low AMR risk) represent at least 60% of overall antibiotic consumption in humans by 2030.

The hope is that these targets will set the stage for concrete commitments at the United Nations General Assembly High-Level Meeting on AMR planned for 2024.

https://bit.ly/3ORIuMx

## A step towards a pandemic accord

WHO Member States agreed to develop the draft of a legally binding agreement designed to help protect the world from future pandemics.

The Intergovernmental Negotiating Body (INB) gathered at WHO headquarters in Geneva, Switzerland from 5–7 December and agreed that the INB’s Bureau, comprised of delegates from each of the six WHO regions, will develop the draft.

Based on a conceptual draft published in November 2022 and the discussions held during the three-day meeting, the draft will serve as the basis for negotiations at the fourth INB meeting, scheduled to start on 27 February 2023.

“The decision to task us with the duty to develop a zero draft of a pandemic accord represents a major milestone in the path towards making the world safer,” said Roland Driece, Co-Chair of the INB Bureau.

https://bit.ly/3FA11tz

## Testing emergency response in Africa

The WHO Regional Office for Africa and partners ran a public health emergency operation centre simulation exercise to step up readiness to respond to public health emergencies in the region.

Played out over 6–7 December, the exercise was the region’s largest to date with 36 participating countries. It simulated the early detection of an Ebola outbreak in a fictitious country and its subsequent spread to multiple countries across the region through international travel and trade.

https://bit.ly/3VZ7SlS

## SARS-CoV-2 variants still a concern

WHO Director-General Tedros Adhanom Ghebreyesus expressed concern that gaps in severe acute respiratory syndrome coronavirus 2 (SARS-CoV-2) surveillance, testing, sequencing and vaccination are “continuing to create the perfect conditions for a new variant of concern to emerge that could cause significant mortality.”

The remark was made at a media conference held on 2 December 2022, just over a year after WHO announced the emergence of the last variant of concern, Omicron. While characterized by a lower mortality rate than its predecessor (Delta), Omicron has proven to be significantly more transmissible and continues to take lives, including 8500 reported deaths in the week prior to the conference.

Dr Tedros noted that, as of 2 December, over 500 sublineages of Omicron were circulating, all highly transmissible, and with mutations that enable them to escape immunity more easily. WHO continues to urge all countries to take a risk-based approach that protects both public health and human rights.

https://bit.ly/3VTGMwh

## Malaria challenges

The global malaria response faced continuing challenges in 2021, threatening progress towards targets set out in the WHO *Global technical strategy for malaria 2016–2030*.

According to the *World malaria report 2022* released on 8 December, disruptions during the COVID-19 pandemic, converging humanitarian crises, health system challenges, restricted funding, rising biological threats and a decline in the effectiveness of core disease-cutting tools were among the main challenges.

To focus on just two, funding for malaria in 2021 was US$ 3.5 billion, well below the estimated US$ 7.3 billion required to achieve global targets, while a decline in the effectiveness of malaria control tools, notably insecticide-treated bednets, is hampering prevention efforts.

https://bit.ly/3Bnr9p5

## Measles vaccination coverage down

In 2021, a record high of nearly 40 million children missed a measles vaccine dose – 25 million missing their first dose and 14.7 million children missing the second. This is according to a joint report by WHO and the United States Centers for Disease Control and Prevention which was released on 23 November.

According to the report, 2021 saw an estimated 9 million measles cases worldwide and 128 000 measles-related deaths. Twenty-two countries experienced large and disruptive outbreaks.

Declines in vaccine coverage, weakened measles surveillance, and continued interruptions and delays in immunization activities due to COVID-19, as well as persistent large outbreaks in 2022, mean that measles is now an imminent threat in every region of the world.

https://bit.ly/3h00Vlv

## Sudan virus outbreak

The outbreak of Sudan virus (a strain of Ebola virus), first declared by Uganda health authorities on 20 September, appeared to be winding down. As of 8 December, no new cases had been reported since 27 November. On 2 December, health authorities reported that all patients had been discharged from Ebola treatment units.

As of 5 December, a total of 142 confirmed cases had been reported by the Uganda Ministry of Health. Fifty-five of the people infected had died. In addition, 22 probable cases had also been reported. Some 2167 of the 2564 contacts listed had completed a 21-day follow-up period, and 36 contacts were still being monitored.

https://bit.ly/3FDessI

**Cover photo**

A nurse gives a child a routine vaccine at a mobile health clinic in Ntiliya village, Marsabit County, Kenya, one of several countries in the greater Horn of Africa facing one of the worst droughts in recent decades.

**Figure:**
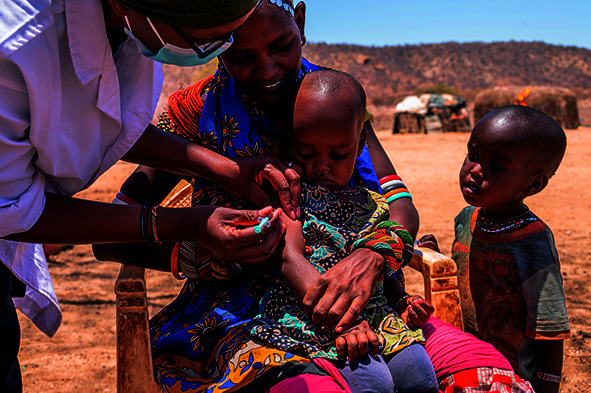
WHO/Billy Miaron

## Preventing injuries and violence

Three of the five leading causes of death of people aged 5–29 years are injury related according to a report released by WHO on 29 November.

Road traffic injuries, suicide and homicide are the main causes of injury-related mortality accounting for roughly 1 in 3, 1 in 6, and 1 in 9 of the 4.4 million annual injury-related deaths, respectively.

The report offers technical guidance on the measures needed to bring those numbers down, while emphasizing the importance of high-level political support and investment in injury prevention measures.

http://bit.ly/3FedoeN

## Disability and exposure to health risks

People living with disabilities are exposed to a higher risk of premature death and illness than people without. This is according to the *Global report on health equity for persons with disabilities*, published by WHO on 2 December.

The report highlights key factors underpinning this disparity, ranging from negative attitudes of some health-care providers towards people with disabilities to lack of access to health facilities due to poor facility design and lack of transport.

The report sets out key interventions to tackle the problem, drawing on the latest evidence from academic studies as well as consultations with countries and civil society, including organizations representing persons with disabilities.

https://bit.ly/3XY5tJY

**Looking ahead**

16–20 January. World Economic Forum Annual Meeting: Cooperation in a Fragmented World. Davos, Switzerland. https://bit.ly/3F4jBIY

27–29 January. The Prince Mahidol Award Conference 2023. Theme: Setting a new health agenda. https://bit.ly/3VWT1rX

30 January–7 February. 152nd Session WHO Executive Board meeting. WHO headquarters, Geneva, Switzerland. https://bit.ly/3uwIick

